# Insurance Proofs and the Attending Physician

**Published:** 1916-11

**Authors:** 


### Insurance Proofs and the Attending Physician.

We have been asked to express an opinion on this subject. The following is presented for what it is worth with the hope that it may be freely criticised.

An insurance company obviously has the right to demand proper proof of death and must depend ultimately on the report of the attending physician. But, it must not be forgotten that the function of insurance is to insure, and that insurance should be paid promptly; that the insurance company has no claim on the services of the attending physician and no right to invade the confidential relations existing between him and his patient. Neither should insurance be voided on account of any technical flaw. For example, a poor widow lost her home by fire and payment of insurance was refused on the ground that an outbuilding stood too near the house, though this had nothing to do with the fire itself. Fortunately, so many threats of cancellation were made by influential friends that the claim was eventually paid. One of the writer's patients was killed by a bullet. Payment of insurance was refused on the ground that a misstatement had been made in the application in regard to the existence of gastric ulcer and the company tried to force a statement—contrary to the principle of privileged communications—to void the insurance. It is perfectly obvious that the question of gastric ulcer had no relation to the cause of death and it is not at all probable that the patient had sufficient technical knowledge of his own case to warrant the belief that he deliberately made any misstatement to the company examiner.

Our solution of the problem would be as follows: Do away entirely with the personal approach of insurance representatives to physicians, undertakers, nurses or friends. Base the proof of death entirely on death and burial reports filed with the proper health department. The government is directly responsible for the accuracy of these records and they are open to inspection by any one properly interested in them, either free or at a nominal charge. While these records are based on the skill and truthfulness of private individuals, the government has, so far as practicable, the power to inforce accuracy and, granting that it has not, it is not probable that a physician will materially change his statements in making out a separate report for the insurance company. Establish a reasonable but brief time limit on the payment of insurance claims, say ten days from the date on which formal notice has been given that the death has occurred and that the proper certificates have been filed with the local health department. Provide for excess rate on any delayed payment enough to discourage delay but not of unreasonable amount, say 1% a month as in the case of delayed taxes. As in the case of contracts in general, the insurance company would, of course, have the right to contest cases in which fraud might be claimed though, if possible, the initiative should rest with the company, from the very nature of insurance. The attending physician, undertaker or anyone else having personal knowledge of the case could, in such an event, be subpoenaed. Serving as witness is a disagreeable task and one that is seldom properly requited but it is a duty of the individual to the community, offset by his own potential rights for the same service. The ordinary legal safeguarding of evidence and especially the principle of privileged communications would thus be insured.

In the case of health and accident insurance, very different procedures would be required, unless in the future, such insurance is to lie entirely in the hands of the state. Under present conditions, it is difficult to avoid securing statements from attending physicians. The best solution of the problem that we can suggest is a law requiring representatives of the company to call upon the patient only in company with the attending physician or such other as the patient may designate, to place a heavy penalty upon the invasion of a patient's room, in a hospital or his own house or elsewhere, by an agent of the company except thus accompanied, to require reasonable promptness in making such a joint visit, to establish a reasonable average fee or schedule of graded fees for the issuance of a certificate by the attending physician, to be paid directly and immediately by the insurance company, and ultimately or not by the patient, from his benefit, according to the contract which, of course, would be subject to competition among different companies.

